# Endoscopic therapy for gastrointestinal bleeding in Klippel–Trénaunay syndrome: a case report and literature review

**DOI:** 10.3389/fmed.2026.1960274

**Published:** 2026-10-15

**Authors:** Ruoxi Zhang, Wen Shi, Xi Wu, Aiming Yang, Yang Chen

**Affiliations:** 1 Department of Gastroenterology, Peking Union Medical College Hospital, Chinese Academy of Medical Sciences and Peking Union Medical College, Beijing, China; 2 Beijing Key Laboratory of Techniques for the Diagnosis and Treatment of Early Gastrointestinal Cancer, Beijing, China

**Keywords:** band ligation, endoscopic treatment, gastrointestinal bleeding, Klippel–Trénaunay syndrome, sclerotherapy, vascular malformations

## Abstract

**Background:**

Klippel–Trénaunay syndrome (KTS) is a rare congenital vascular anomaly characterized by capillary malformation, underlying low-flow venous/lymphatic malformations, and bone- and/or soft-tissue limb hypertrophy of the affected extremity. At present, there is no consensus on the optimal management of KTS, particularly in the context of gastrointestinal bleeding.

**Case presentation:**

We describe a case of gastrointestinal bleeding in a patient with KTS who presented with colorectal venous malformations with phleboliths, multiple splenic vascular malformations, penile varicosities, and hypertrophy of the right lower extremity and the right buttock. A colonoscopy revealed a circumferential mucosal elevation with bluish discoloration extending from 15 cm above the anal verge in the colorectum, and active bleeding was observed at 12 cm from the anal verge. Endoscopic submucosal sclerotherapy with 1–1.5 mL of lauromacrogol (1%) per site and a total volume of 30 mL, combined with band ligation using six bands, was performed. This approach has rarely been reported and resulted in significant relief from bleeding. At the 6-month follow-up, the patient's symptoms and hemoglobin levels remained stable, with no other complications.

**Conclusion:**

Although KTS remains incurable, early endoscopic recognition facilitates diagnosis, and endoscopic therapy may serve as an important treatment modality for gastrointestinal involvement.

## Background

Lower gastrointestinal bleeding is a common clinical problem with a broad differential diagnosis. Although hemorrhoids, diverticular disease, colorectal neoplasms, and inflammatory bowel disease account for most patients with this condition, a small but important proportion of patients have rare vascular causes, including gastrointestinal vascular malformations (VMs). These lesions may present with overt bleeding, such as hematochezia or melena, but they more frequently manifest as occult or recurrent bleeding, leading to chronic iron-deficiency anemia and diagnostic delay. A high index of suspicion for vascular anomaly syndromes is particularly warranted in young patients, those with recurrent gastrointestinal bleeding, and those with vascular anomalies or soft-tissue overgrowth in other regions. Early recognition and diagnosis facilitate more targeted treatment and help avoid adverse outcomes such as massive hemorrhage caused by blind biopsy.

Klippel–Trénaunay syndrome (KTS) is a congenital mesodermal disorder characterized by the classic triad of varicose veins, capillary malformations, and bone- and/or soft-tissue hypertrophy and has an estimated incidence of 1 per 100,000 people globally (1). This complex overgrowth condition is distinguished from other overgrowth syndromes by the presence of slow-flow malformations and the absence of arteriovenous malformations (AVMs). Diagnosis is based on the 2018 International Society for the Study of Vascular Anomalies (ISSVA) criteria, which require the presence of capillary malformations, venous malformations, and bone- and/or soft-tissue overgrowth in an affected limb. Recent evidence indicates that somatic mosaic mutations in PIK3CA, leading to the hyperactivation of the PI3K-AKT-mTOR pathway, are the major molecular mechanism underlying KTS, which places it within the PIK3CA-related overgrowth spectrum (PROS) (2).

Gastrointestinal involvement occurs in less than 20% of patients with KTS, most commonly affecting the rectum and sigmoid colon (3, 4). Manifestations typically include colorectal edema and mural thickening, resulting from VMs located within and adjacent to the bowel wall. These abnormal vascular structures frequently cause recurrent rectal hemorrhage, ranging from subtle occult bleeding to life-threatening massive episodes, with varying degrees of anemia (5). At present, no consensus exists regarding the optimal management of KTS. Most patients with KTS-related gastrointestinal bleeding receive partial bowel resection (6, 7) or conservative treatment (3, 8–10). However, with advances in gastrointestinal endoscopy, endoscopic treatment procedures such as sclerotherapy and argon laser coagulation have been reported in recent years (11, 12). Nevertheless, the overall clinical experience remains limited. We report a case of KTS presenting with severe lower gastrointestinal bleeding and describe the diagnostic and therapeutic course, aiming to provide novel insights into the management of this rare condition.

## Case presentation

A 59-year-old man presented with chronic gastrointestinal bleeding since birth, manifesting as fresh blood on the surface of the stool, which had markedly worsened over the past year with severe anemia. The lowest hemoglobin level was 40–50 g/L, and therefore, the patient required intermittent blood transfusions. The patient had a history of impaired mobility of the right lower extremity since childhood. His family has no history of genetic conditions or malignancies. A physical examination revealed hypertrophy of the right buttock and the lower limb, multiple subcutaneous varicose veins with calcified nodules on the right lower extremity, penile varicosities, and splenomegaly (Figures 1A–C). A digital rectal examination identified nodular elevations of the rectal wall, with no blood observed.

**Figure 1:**
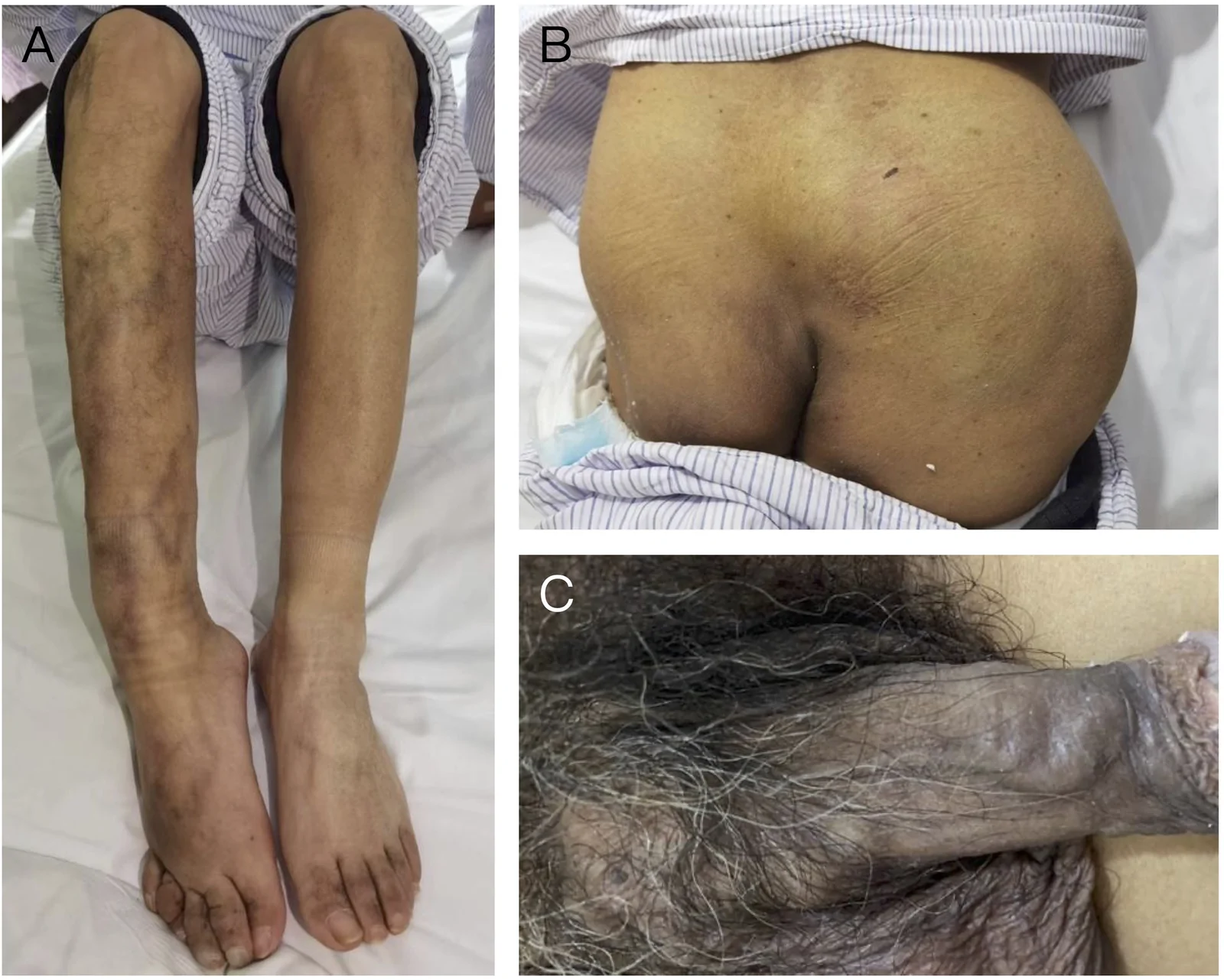
Cutaneous VMs and hypertrophy of the right lower limb. **(A)** Multiple dilated and tortuous subcutaneous venous varicosities in the right lower extremity and hypertrophy of the right lower limb. **(B)** Marked enlargement of the right buttock. **(C)** Subcutaneous venous varicosities on the penis.

MRI results demonstrated VM-like changes in the rectal and sigmoid colonic walls with secondary luminal stenosis (Figure 2A). Abdominal contrast-enhanced CT showed rectal and sigmoid colonic wall thickening with multiple calcifications; calcifications in the bilateral adrenal glands, pelvis, and scrotum (Figures 2B,C); multiple tortuous nodular densities with internal calcification in the subcutaneous tissue of the right buttock; splenic hemangiomas (the largest one measuring approximately 4.5 × 5.0 cm); and the mildly dilated portal vein (1.3 cm) and splenic vein (1.8 cm, Figure 2D). Ultrasound imaging showed organized intramuscular venous thrombosis with phlebolith formation in the right lower extremity. Lymphoscintigraphy showed no abnormalities. Gastroscopy revealed no gastroesophageal varices.

**Figure 2:**
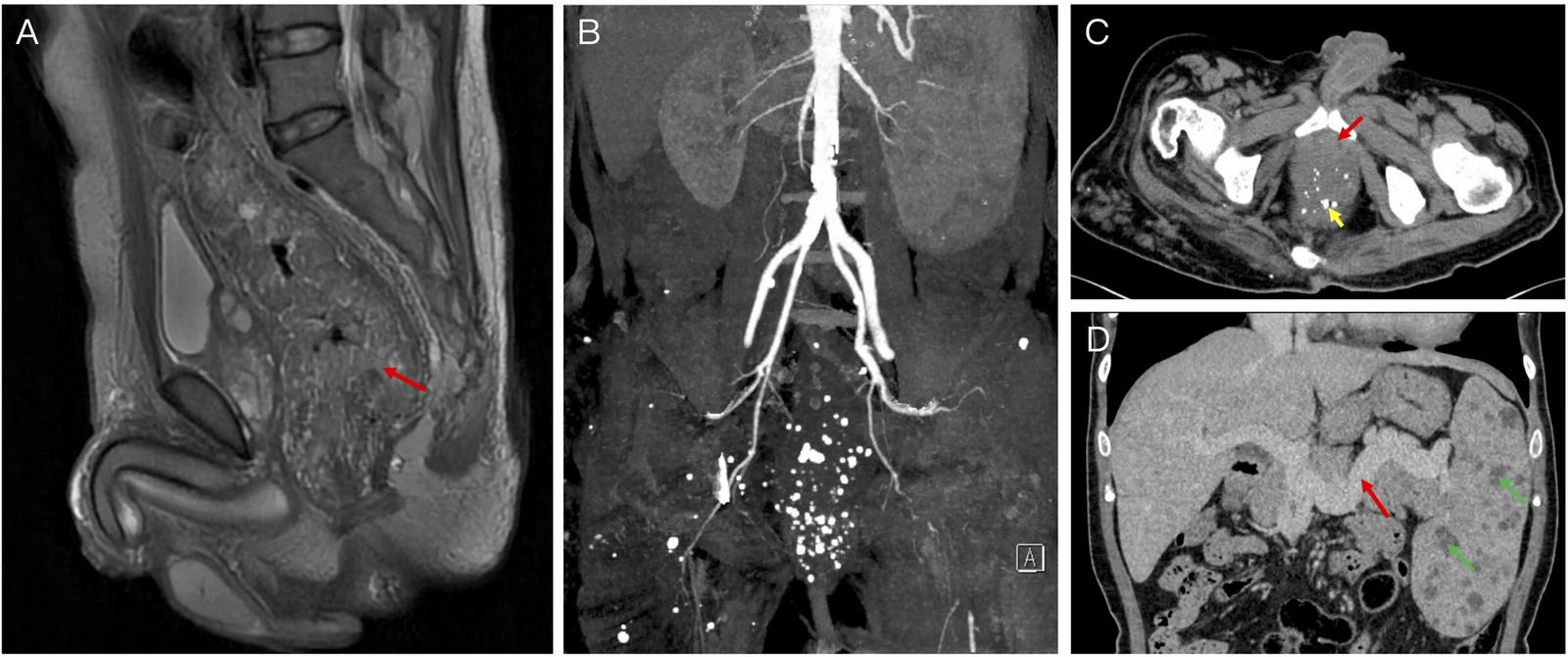
Abdominopelvic MRI and CT scans. **(A)** An MRI scan demonstrates VM-like changes in the rectal and sigmoid colonic walls, which is indicated by the red arrow. **(B)** A CT scan shows multiple phleboliths in the rectal and sigmoid colonic walls, the right lower extremity, and the gluteal region. **(C)** A CT scan reveals thickened rectal wall with phleboliths (indicated by the yellow arrow) throughout all layers (indicated by the red arrow). **(D)** Splenomegaly and splenic VMs (indicated by the green arrow); portal vein reconstruction demonstrates dilation of the splenic vein (indicated by the red arrow) and mild dilation of the portal vein.

Colonoscopy revealed a circumferential mucosal elevation with bluish discoloration extending from 15 cm above the anal verge in the colorectum (Figures 3A,B). Vascular ectasia with active bleeding was observed at 12 cm from the anal verge (Figure 3C). There were multiple smooth-surfaced nodular elevations resembling clusters of grapes at 5 cm from the anal verge (Figure 3D). Endorectal ultrasound imaging revealed that the submucosal layer was thickened and hypoechoic, with some areas showing fine honeycomb-like changes and blood flow signals on a color Doppler scan, which was suggestive of VMs (Figure 3E).

**Figure 3:**
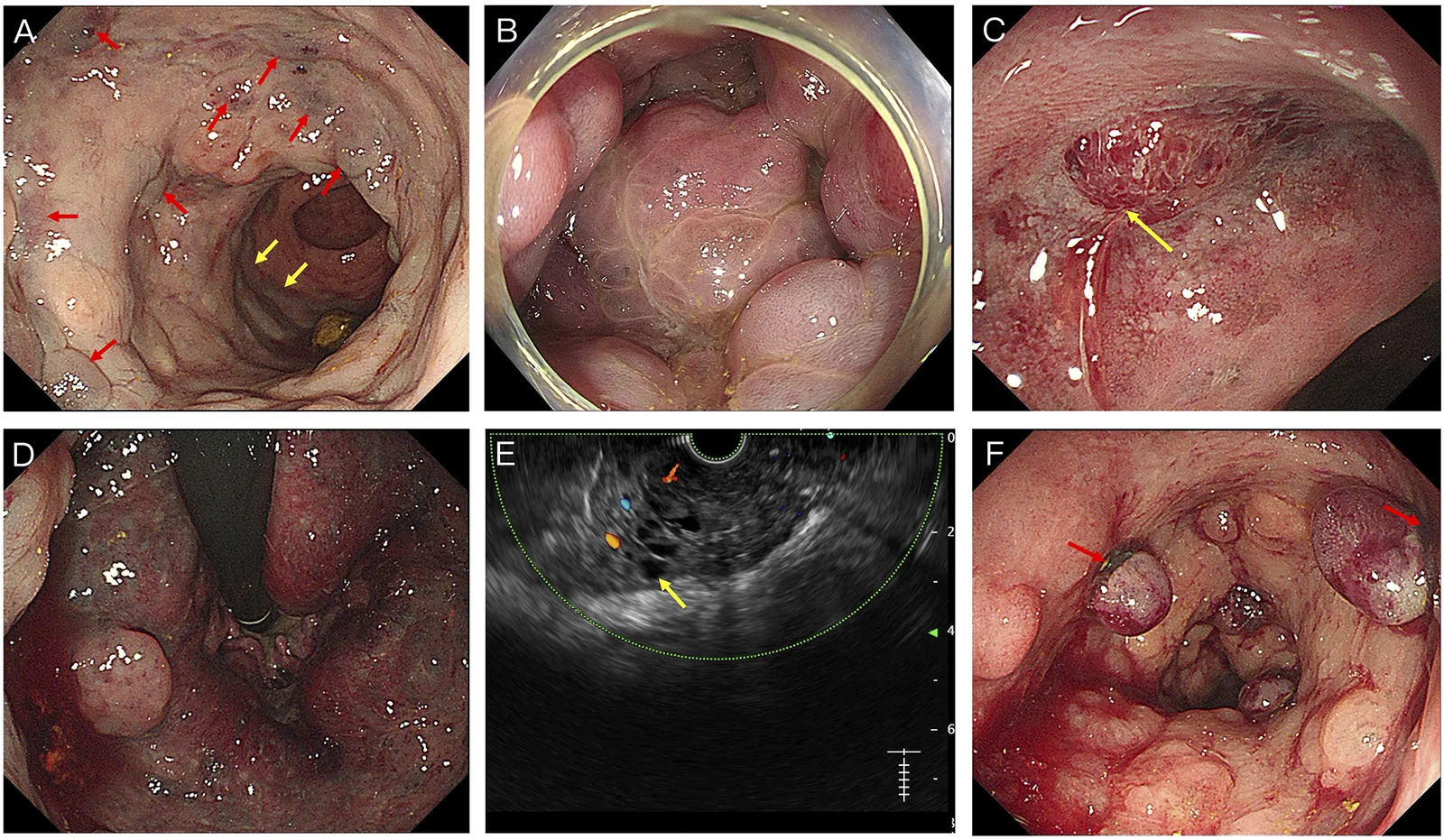
Endoscopic findings and treatment. **(A)** The yellow arrows indicate the oral margin of the lesion at 15 cm from the anal verge, whereas the red arrows indicate multiple flat elevations with mucosal hyperemia and vascular dilation. **(B)** A colonoscopy revealed multiple nodular protrusions with a grape-like appearance in the rectum. **(C)** The yellow arrows indicate active bleeding at 12 cm from the anal verge. **(D)** The lesion extended to the anal verge with multiple nodular protrusions. **(E)** An endorectal ultrasound scan revealed that some areas of the affected bowel segment showed fine honeycomb-like changes, with blood flow signals on a color Doppler scan. **(F)** Appearance after treatment. The red arrows indicate two of the ligation sites.

Initial evaluation focused on common causes of lower gastrointestinal bleeding, all of which were excluded based on colonoscopy findings. CT, MRI, and endoscopic ultrasound findings showed phleboliths and low-flow VMs without an arterial component, suggesting that the gastrointestinal bleeding was most likely due to venous malformations. These findings along with the childhood onset and systemic manifestations, including multiple splenic VMs, subcutaneous and penile varicosities, and hypertrophy of the right lower extremity and the right buttock, supported the diagnosis of KTS.

Because surgical resection would have precluded sphincter preservation, local endoscopic therapy was cautiously performed after multidisciplinary team discussion (MDT), with due consideration to the quality of life and patient preference. The previously identified bleeding site at 12 cm from the anal verge was treated with submucosal injection of lauromacrogol using a 23-gauge injection needle (1%; 1–1.5 mL per site and a total volume of 30 mL) at multiple sites around the bleeding site and at the base of the venous malformations. Six bands were then deployed in a spiral configuration around the bleeding site, targeting markedly elevated and erythematous areas at high risk for bleeding, to interrupt the lesional blood flow (Figure 3F). Immediate hemostasis was achieved, and no active bleeding was observed at the end of the procedure. No procedure-related complications occurred. Adjunctive therapy included hemostatic agents, iron supplementation, and blood transfusions. Given the considerations of cost, drug accessibility, and potential adverse effects, the patient did not receive systemic medical therapy. In addition, the patient declined splenectomy. The patient experienced no further active bleeding, and his hemoglobin levels increased to 105 g/L at the 3-month follow-up. At the 6-month follow-up, the patient reported no obvious melena or hematochezia, and intermittent monitoring showed that his hemoglobin level fluctuated between 100 and 110 g/L.

## Discussion

KTS is a rare congenital disorder with multisystem involvement. Affected patients may experience a variety of complications that adversely impact their quality of life and overall health. No universally accepted gold standard exists for the diagnosis of KTS. The 2018 ISSVA diagnostic criteria encompass capillary malformations, venous malformations, and bone- and/or soft-tissue overgrowth in an affected limb. In clinical practice, diagnosis may also be established through a combination of imaging evaluations, angiography, and genetic testing, often serving as a diagnosis of exclusion to rule out other conditions.

Accumulating evidence suggests that mosaic somatic mutations in the PIK3CA gene are the most significant molecular etiology of KTS (2). The phosphoinositide-3 kinases (PI3Ks) are key lipid kinases that control signaling pathways involved in cell proliferation, motility, survival, and metabolism. The PIK3CA gene encodes the p110α catalytic subunit of the PI3K lipid kinase. Therefore, mutations in this gene lead to structural alterations in p110α that prevent it from binding to the p85α regulatory subunit, which normally suppresses its activity (13). This results in hyperactivation of the PI3K-AKT-mTOR pathway, leading to dysregulated cell proliferation and consequent segmental overgrowth of tissues such as blood vessels, soft tissues, and bone. Despite their frequent involvement in oncogenesis, PIK3CA variants do not appear to increase the risk for malignancy in patients with PROS diseases (13). However, in addition to PIK3CA, KTS has been reported in association with chromosomal anomalies, including balanced translocations (14, 15) and a supernumerary chromosome 18 (16); genetic defects of VG5Q including chromosomal translocation *t*(5;11) and mutated E133 K (17); and terminal deletion 2q37.3 (18).

For patients presenting with gastrointestinal involvement, particularly those with lower gastrointestinal bleeding as the predominant manifestation, common causes of bleeding such as hemorrhoids, neoplasms, diverticular disease, and inflammatory bowel disease should be excluded first. Endoscopy plays a crucial role in the initial identification and differentiation of vascular lesions, as biopsy of VMs may lead to life-threatening hemorrhage, and it can also define the location and extent of VMs throughout the gastrointestinal tract. Notably, some gastrointestinal manifestations of KTS may closely mimic ulcerative colitis (19), with overlapping symptoms and endoscopic findings, including recurrent bloody diarrhea and continuous circumferential involvement of the distal colon with bleeding. These overlapping features highlight the need to consider KTS as a multisystem disorder, especially when gastrointestinal symptoms coexist with VMs and limb abnormalities.

Once gastrointestinal VMs are identified, the differential diagnosis should be broadened to include other vascular anomalies. In VMs, determining the involved vascular components and assessing the extent of involvement are essential. CT is one of the most important imaging modalities for this purpose, as it can reveal phleboliths and transmural wall thickening of the affected bowel segment (20). However, because of the relatively quiescent and stagnant flow within these malformative veins, contrast entry into the lesions is limited and diffusion is poor. Therefore, these lesions may not be adequately enhanced or clearly demarcated in CT imaging. MRI is particularly sensitive for evaluating venous malformations, vascular dilatation, and blood reflux (21). Moreover, dynamic postcontrast MRI sequences can help distinguish high-flow from low-flow vascular lesions, with high-flow lesions showing rapid filling (22).

In the diagnostic evaluation of patients with childhood-onset chronic gastrointestinal bleeding, a detailed history, physical examination, and imaging evaluations are essential. In young patients with multiple VMs, a high degree of suspicion for an underlying syndromic disorder is warranted. For example, Proteus syndrome (PS) shares several features with inverse KTS, including patchy areas of undergrowth and reduced skin and adipose tissue, but it is also characterized by disproportionate overgrowth, connective tissue and epidermal nevi, and dysregulated adipose tissue (23). Detection of a somatic-activating AKT1 c. 49 G>A p. E17 K variant supports a diagnosis of PS (24). Parkes Weber syndrome (PWS) represents the closest mimic of KTS, but it can be distinguished by its arteriovenous fistulas, limb hypertrophy, and ipsilateral cutaneous hemangioma; RASA1 mutations favor PWS (25). CLOVES syndrome also falls within the PROS, yet it differs from KTS by the presence of manifestations such as congenital lipomatous overgrowth, epidermal nevi, and scoliosis/skeletal/spinal anomalies (26). Importantly, high-flow AVMs, which occur in CLOVES and PWS, have not been described in KTS, further highlighting the importance of ultrasound, MRI, and CT imaging in differentiating arteriovenous malformations from venous malformations. In addition, truncal involvement is typically absent in KTS, with anomalies predominantly affecting the limbs. In our patient, childhood onset, colorectal venous malformations with phleboliths, multiple splenic VMs, penile and subcutaneous varicosities, and hypertrophy of the right lower extremity and the right buttock—together with the absence of arterial involvement on comprehensive imaging—supported a final diagnosis of KTS.

At present, no definitive cure exists for KTS. Therefore, treatment is predominantly conservative and symptomatic, with the principal aim of enhancing the quality of life of the patient. Given the multisystem involvement of KTS, a MDT approach is generally recommended. Most reported cases of patients with KTS along with gastrointestinal tract involvement have been managed conservatively (3, 8–10) or via surgical resection (6, 7). Surgical resection procedures, such as sphincter-preserving excision of the affected sigmoid colon and rectum followed by a colon pouch anal anastomosis and protective loop ileostomy, should be considered in patients with severe symptoms and extensive involvement. However, in patients with a rectal lesion that is located too low for sphincter-preserving surgery, local endoscopic therapy may be considered.

Endoscopic therapies, especially sclerotherapy, have been increasingly applied to gastrointestinal VMs (27, 28). These modalities have become first-line treatment options for selected VMs, such as angiodysplasia and Dieulafoy's lesion (20, 29). Sclerotherapy induces endothelial injury, thrombosis, and luminal occlusion through chemical stimulation, whereas endoscopic laser therapy coagulates abnormal vessels under endoscopic guidance. Commonly used sclerosants include lauromacrogol, sodium tetradecyl sulfate, ethanolamine oleate, cyanoacrylate glue, hypertonic glucose, and absolute ethanol. However, endoscopic treatment for KTS has been documented only in isolated case reports, including sclerotherapy (11, 12) and endoscopic argon laser coagulation (19) (Table 1). Nevertheless, these approaches have shown favorable efficacy in controlling gastrointestinal bleeding. Endoscopic band ligation has been rarely reported in KTS and may also be considered as an option in combination with sclerotherapy. In our case, local endoscopic therapy alleviated the symptoms of gastrointestinal bleeding, suggesting that it may be a potential treatment option for gastrointestinal bleeding in KTS, although additional evidence from clinical trials is still required to confirm its efficacy. To the best of our knowledge, this is the first reported case of KTS with extensive anorectosigmoid venous malformations treated by a combination of endoscopic lauromacrogol injection and band ligation.

**Table 1:** Summary of the patients reported with KTS gastrointestinal involvement and endoscopic therapy.

Reference	Sex	Age	Symptoms	Gastrointestinal lesions	Endoscopic therapy	Outcome
Wang et al. (11)	Male	32	Intermittent rectal bleeding for 32 years, superficial venous varicosities, swelling in the right lower limb, and vascular abnormalities in the liver	Multiple venous varicosities from the splenic flexure to the rectum	Multiple injections of polidocanol and meilan (45 mL initially and 40 mL at 6 months)	Recurrent rectal bleeding 6 months later; no rectal bleeding and stable hemoglobin level at the 1-year follow-upNo complications reported
Agostinho et al. (12)	Male	18	Multiple significant episodes of hematochezia	Colonic vascular malformations, non-obstructive pedunculated malformation near the ileocecal valve, and diffuse-dilated rectal venous plexus	Endoscopic sclerotherapy: 5 mL phenol in almond oil (left rectum) + 20 mL 75% ethanol (right-sided vessels) initially; at 6 months, 20 mL 75% ethanol (left) + 10 mL 75% ethanol (right)	No further hematochezia reported; subjective reduction in right-sided lesions (ethanol-injected) more than those in the left No complications reported
AlAli et al. (19)	Male	64	Recurrent foul-smelling bloody diarrhea for 2 months; severe iron deficiency anemia (hemoglobin, 65 g/L)	Active bleeding and extensive venous dilations extending 15 cm from the anal verge	Endoscopic argon laser coagulation of the vascular lesions	Ongoing follow-up No complications reported

However, in anorectosigmoid VM, the venous drainage of the upper rectum and the sigmoid colon flows into the inferior mesenteric vein (IMV), which belongs to the portal venous system. Dilated and incompetent IMV have been previously reported in colorectal VM (30). Therefore, concerns have been raised that when sclerotherapy is used to treat anorectosigmoid VM in patients with KTS, the sclerosants themselves and the induced thrombi may migrate into the portal system via the IMV (21, 30). This process could potentially induce or exacerbate *de novo* portal vein thrombosis and/or portal hypertension, thereby worsening anorectosigmoid VM and bleeding. Although this hypothesis requires further clinical confirmation, it implies that prolonged close monitoring is necessary after sclerotherapy (whether endoscopic or interventional), especially in patients with portal hypertension.

As the molecular mechanisms underlying KTS are better understood now, targeted therapeutic options have emerged. In 2022, alpelisib, a p110α inhibitor, became the first medical therapeutic drug to receive US Food and Drug Administration (FDA) approval for PROS, with efficacy demonstrated in the retrospective EPIK-P1 study (31). It has an acceptable safety profile; however, lifelong therapy may be required. Rapamycin, also known as sirolimus, is an allosteric inhibitor of mTOR. Low-dose sirolimus has been shown to moderately reduce overgrowth; however, its relatively pronounced adverse-effect profile mandates individualized risk–benefit assessment (32). The VASE multicenter study, an ongoing phase 3 clinical trial, has reported that individualized, intermittent sirolimus regimens can effectively control symptoms in patients with PIK3CA-related VMs and significantly reduce adverse events (33). It may also be effective in VMs even in the absence of a genetic diagnosis (34). Apart from drug-based interventions, supportive measures, encompassing psychological care, are of comparable importance. The clinical course is usually favorable, especially in younger patients, but it may be severe in those with certain related underlying conditions. Close surveillance for gastrointestinal bleeding from VMs is essential, as such bleeding may be occult and chronic and can cause severe anemia.

## Conclusion

In conclusion, early recognition and timely treatment of KTS are of particular importance in reducing and alleviating KTS-related complications. For patients with KTS along with gastrointestinal involvement, local endoscopic therapy may be considered, although further large-scale studies are warranted.

## Data Availability

The raw data supporting the conclusions of this article will be made available by the authors, without undue reservation.
